# Supplementary material for: Resolving the full spectrum of human genome variation using Linked-Reads
Source: Genome Res. 2019 Apr;29(4):635–45. doi: 10.1101/gr.234443.118 (PMC6442396; doi:10.1101/gr.234443.118)
Supplement: Supplemental Material [file supp_gr.234443.118_Supplemental_longranger-2.2.zip › longranger-2.2.tar.bz2!/lr2.2-public/lib/stan/cmdstan/stan/doc/api/html/base__integrator_8hpp_source.html]

Stan: src/stan/mcmc/hmc/integrators/base\_integrator.hpp Source File


|  |  |
| --- | --- |
|  | Stan  2.5.0  probability, sampling & optimization |


- Main Page
- Related Pages
- Namespaces
- Classes
- Files

- File List
- File Members

All Classes Namespaces Files Functions Variables Typedefs Enumerations Enumerator Friends Macros Pages

- src
- stan
- mcmc
- hmc
- integrators

base\_integrator.hpp

Go to the documentation of this file.

1 #ifndef STAN\_\_MCMC\_\_BASE\_\_INTEGRATOR\_\_BETA

2 #define STAN\_\_MCMC\_\_BASE\_\_INTEGRATOR\_\_BETA

3

4 #include <ostream>

5

6 namespace stan {

7

8  namespace mcmc {

9

10  template <typename H, typename P>

11  class base\_integrator {

12

13  public:

14

15  base\_integrator(std::ostream\* o): out\_stream\_(o) {};

16

17  virtual void evolve(P& z, H& hamiltonian, const double epsilon) = 0;

18

19  protected:

20

21  std::ostream\* out\_stream\_;

22

23  };

24

25  } // mcmc

26

27 } // stan

28

29

30 #endif

stan::mcmc::base\_integrator::base\_integrator

base\_integrator(std::ostream \*o)

**Definition:** base\_integrator.hpp:15

stan::mcmc::base\_integrator::out\_stream\_

std::ostream \* out\_stream\_

**Definition:** base\_integrator.hpp:21

stan::mcmc::base\_integrator

**Definition:** base\_integrator.hpp:11

stan::mcmc::base\_integrator::evolve

virtual void evolve(P &z, H &hamiltonian, const double epsilon)=0


---

[ Stan Home Page ]

*© 2011–2014,
Stan Development Team.*
